# Positron emission tomography imaging of endogenous mu-opioid mechanisms during pain and migraine

**DOI:** 10.1097/PR9.0000000000000769

**Published:** 2019-08-07

**Authors:** Alexandre F. DaSilva, Jon-Kar Zubieta, Marcos F. DosSantos

**Affiliations:** a Headache & Orofacial Pain Effort (H.O.P.E.), Department of Biologic and Materials Sciences, University of Michigan School of Dentistry, Ann Arbor, MI, USA; b Department of Psychiatry, University of Utah Health, Salt Lake City, UT, USA; c Instituto de Ciências Biomédicas (ICB), Universidade Federal do Rio de Janeiro (UFRJ), Rio de Janeiro, Brazil

**Keywords:** Pain, Chronic pain, PET, Neuroimaging, Opioids

## Abstract

The enormous advancements in the medical imaging methods witnessed in the past decades have allowed clinical researchers to study the function of the human brain in vivo, both in health and disease. In addition, a better understanding of brain responses to different modalities of stimuli such as pain, reward, or the administration of active or placebo interventions has been achieved through neuroimaging methods. Although magnetic resonance imaging has provided important information regarding structural, hemodynamic, and metabolic changes in the central nervous system related to pain, magnetic resonance imaging does not address modulatory pain systems at the molecular level (eg, endogenous opioid). Such important information has been obtained through positron emission tomography, bringing insights into the neuroplastic changes that occur in the context of the pain experience. Positron emission tomography studies have not only confirmed the brain structures involved in pain processing and modulation but also have helped elucidate the neural mechanisms that underlie healthy and pathological pain regulation. These data have shown some of the biological basis of the interindividual variability in pain perception and regulation. In addition, they provide crucial information to the mechanisms that drive placebo and nocebo effects, as well as represent an important source of variability in clinical trials. Positron emission tomography studies have also permitted exploration of the dynamic interaction between behavior and genetic factors and between different pain modulatory systems. This narrative review will present a summary of the main findings of the positron emission tomography studies that evaluated the functioning of the opioidergic system in the context of pain.

## 1. Introduction

**Figure 1.**
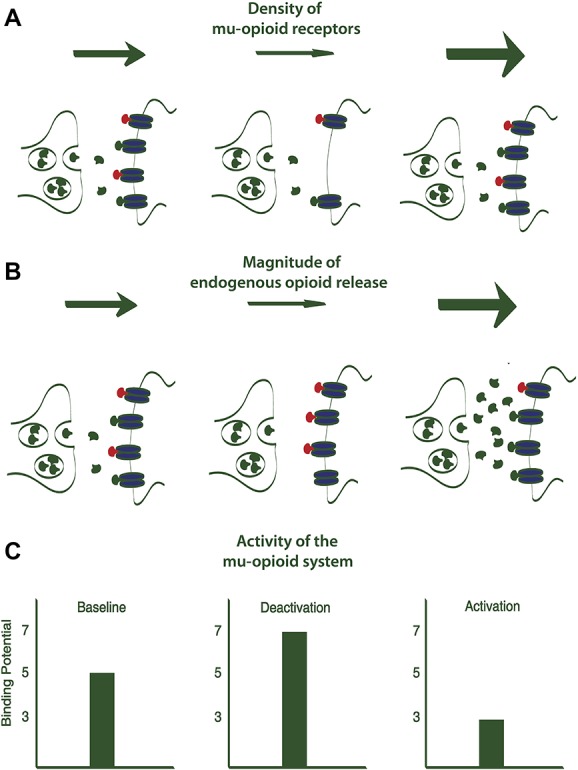
Changes in the binding potential (BP) of mu-opioid receptors could indicate either an altered density of mu-opioid receptors (A) or changes in the magnitude of endogenous opioids release (B), which directly correlates to the activity of the mu-opioid system (C). Presynaptic and postsynaptic membranes are represented in green under different conditions. Mu-opioid receptors are represented in blue. Endogenous opioids released in the synaptic cleft are depicted in green. Mu-opioid receptor agonists (eg, carfentanil) are showed in red.

Positron emission tomography (PET) combines radioactive tracers and mathematical models to determine the kinetics of bioactive tracers involved in certain physiological phenomena. With this method, the measurement of a certain radiotracer concentration in the nervous tissue is represented through a 3-dimensional image, which ultimately represents the anatomical distribution of the biological process under evaluation. Positron emission tomography has allowed researchers to quantify the availability of several receptors in vivo. The receptors studied include serotonergic, dopaminergic, and opioid, among various others, which can be evaluated either in baseline conditions (eg, at rest) or during specific challenges, such as cognitive tasks, experimental pain, or even the administration of a placebo.^39,57,70–75^ Moreover, with this method, it has been possible to study the dynamic functioning of some of the major endogenous pain modulatory systems (eg, dopaminergic and opioidergic) and their interactions in different neuropsychological disorders, including depression,^32^ addiction,^47^ and chronic pain.^25^ For instance, an early PET study conducted in patients with chronic pain revealed that fibromyalgia, a disease with unknown pathophysiology and challenging clinical management, is associated with a decreased availability of mu-opioid receptors, the main target of opiate analgesics, in several pain-related regions including the amygdala, nucleus accumbens (NAc), and cingulate cortex.^25^ Interestingly, a comparable pattern of mu-opioid receptor activation had been demonstrated earlier in a group of healthy volunteers subjected to experimental sustained pain.^73^ Those initial PET studies, which used a radioligand that selectively binds mu-opioid receptors, provided novel information at that time regarding the endogenous regulation of the pain experience by the opioid system. The changes observed in brain areas related not only to the sensory but also to the emotional and cognitive aspects of pain and also corroborated the concept of pain as a multidimensional experience, introduced many years before.^41^ Those findings were further expanded to other chronic pain conditions, such as trigeminal neuropathic pain^19^ and migraine headache.^16,45^ Although those studies used the selective mu-opioid receptor radioligand [11C] carfentanil, similar findings have been obtained using the nontype selective opioid receptor radiotracer [11C] diprenorphine, which has also been largely used to study the role of the whole opioid system in chronic pain syndromes. For instance, increased availability of opioid receptors in the frontal, temporal, and cingulate cortices was associated with decreases in the inflammatory pain of patients with rheumatoid arthritis in a past study.^28^ Another study with [11C] diprenorphine described the presence of reduced opioid receptor availability in both cerebral hemispheres of patients diagnosed with peripheral neuropathic pain.^68^ Of note, in patients with neuropathic pain, the decreased availability of opioid receptors was predominantly located in the hemisphere contralateral to the reported pain.^37^ Such findings could indicate a higher release of endogenous opioid peptides (eg, enkephalins) driven by pain, resulting in higher occupancy and lower availability of opioid receptors (Fig. 1), or alternatively, a downregulation or even loss of opioidergic neurons, with both scenarios associated with a prolonged pain experience. This specific question has been recently addressed in an experimental model study^62^ and will be discussed later in this article. The results obtained in PET studies, from a clinical perspective, might help to explain the lack of efficacy of opioid analgesics, a class of drugs that has been applied in the treatment of cancer and postsurgical pain^13,27^ but usually fails to provide adequate relief in other pain syndromes, including fibromyalgia and chronic neuropathic pain.^23^ Based on these findings, it has been hypothesized that each chronic pain condition may be related to specific changes in the opioidergic system. Therefore, it is important to consider the effects of selected genetic polymorphisms on individual differences in the opioid system response to pain and placebo treatments. These differences have been investigated through PET and will be discussed in this article.

## 2. Opioid system activity related to pain

Pain has been described as “an unpleasant sensory and emotional experience associated with actual or potential tissue damage, or described in terms of such damage,”^43^ a definition that derives from the concept the multidimensionality of pain. This fundamental concept, initially introduced in the 1960s^42^ is still extremely useful to understand the complexity of the pain experience, which goes far beyond nociception (sensory dimension), with its important affective (motivational) and cognitive (evaluative) dimensions. However, with the enhancement of neuroimaging techniques, it has been possible to corroborate and consolidate this model. Magnetic resonance imaging (MRI) studies have provided valuable information regarding functional changes (eg, altered connectivity, metabolism, and hemodynamics) as well as structural changes (cortical thickening, thinning, or altered volume) that occur in the brain driven by pain. In addition, some previous PET studies showed increases in the regional cerebral blood flow related to noxious thermal stimuli of different intensities when compared with innocuous thermal stimulation. The results of such studies suggest that changes in the regional cerebral blood flow associated with noxious but not to non-noxious thermal stimulation could represent neuronal changes associated with nociceptive processing driven by pain.^11^ Other PET studies have confirmed the presence of changes in cerebral blood flow induced by noxious stimuli and have even revealed the brain regions that contribute to each aspect of the pain experience. For instance, 1 PET study that evaluated the effects of noxious heat stimulation on the regional cerebral blood flow confirmed the major role of the thalamus in the processing of pain intensity. However, according to the results of that study, pain unpleasantness may be more associated with the activity of the posterior part of the anterior cingulate cortex (ACC).^63^ Nonetheless, these findings added limited, although extremely relevant, data regarding the activity of specific neurotransmitters and modulatory systems related to pain processing and modulation. With this respect, PET studies that applied selective or nonselective radiotracers to investigate the functioning of particular modulatory systems related to noxious stimulation have contributed significantly to the study of pain, providing important insights into the molecular mechanisms underlying the pain experience as well as the placebo analgesia and the nocebo effect.

In one the first PET studies that used the selective mu-opioid receptor radiotracer [11C] carfentanil, Bencherif et al. demonstrated a decreased mu-opioid receptor availability in the contralateral thalamus related to a noxious stimulus (eg, capsaicin, applied to the dorsal aspect of the hand of healthy volunteers). Interestingly, this decrease in mu-opioid receptor availability in the thalamus directly correlated with the subjective pain ratings. These results suggested a release of endogenous opioids driven by acute pain. They were also important to demonstrate, in vivo, the involvement of the mu-opioid neurotransmission in the supraspinal regulation of pain perception.^8^ In a later study, Zubieta et al.^72^ investigated the activity of mu-opioid receptor-mediated neurotransmission in vivo during a sustained pain challenge, obtained through a controlled injection of 5% hypertonic saline or a placebo into the masseter muscle of healthy volunteers. The results demonstrated the activation of the mu-opioid system associated with an ongoing pain experience. Such activation was represented by a lower availability of mu-opioid receptors during the pain challenge when compared with the placebo phase. Strikingly, those changes were found in a wide variety of pain-related brain structures, such as the amygdala, thalamus, hypothalamus, insula, prefrontal cortex (PFC), and dorsal ACC. These findings served as a basis to further PET studies, and consequent investigation ultimately confirmed the same pattern of mu-opioid activation in patients with different pain syndromes.^16,19,25,45^ Interestingly, a recent PET/MRI study demonstrated that the mu-opioid system is not only involved in self-experienced pain but also regulates the neural mechanisms that trigger the unpleasant sensation of seeing others in pain, namely vicarious pain (an essential component of the human behavior). More precisely, a negative correlation between hemodynamic changes (eg, blood oxygen level dependent [BOLD] signal) and mu-opioid receptor availability was detected in the insula (anterior and posterior), thalamus, primary motor cortex (M1), primary and secondary somatosensory cortex (S2), paracentral lobule, supplementary motor area, and PFC. Given these findings, these brain regions appear to process the negative features of pain as well as play a role in the sensory-discriminative mirroring of others' pain. On the other hand, the availability of mu-opioid receptors in the orbitofrontal cortex (OFC) was positively correlated to its BOLD activation induced by vicarious pain, which could possibly be linked to a role of this brain region in socioemotional functions and mentalizing. Importantly, the involvement of dopamine D2 receptors could not be demonstrated in that study.^29^

**Figure 2.**
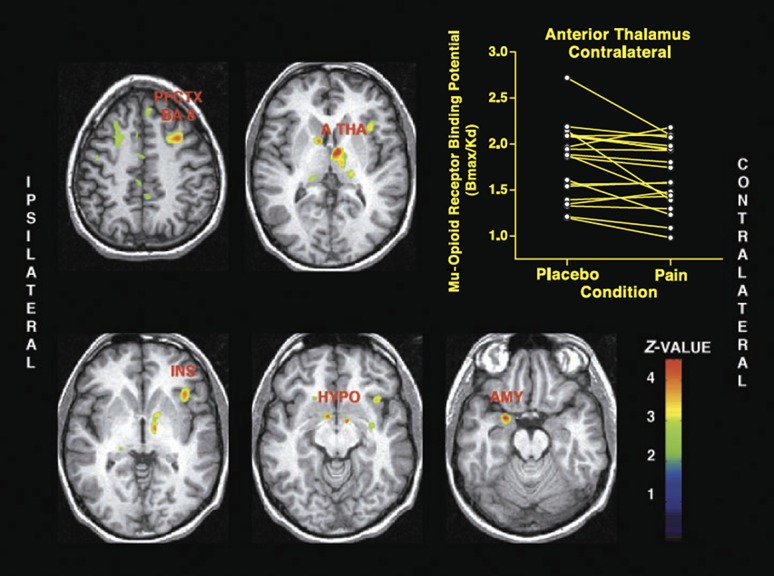
Mu-opioid system activation during sustained masseter muscle pain. Brain regions where significant decreases in the nondisplacable binding potential (BP_ND_), meaning lower mu-opioid receptor availability, from placebo to sustained pain are illustrated on the left. The scatter plot with the respective values in the thalamus is presented on the right. PFCTX BA 8, prefrontal cortex, Brodmann areas 8/9; A THA, anterior thalamus; INS, anterior insular cortex; HYPO, hypothalamus; AMY, amygdala.^72^ From Zubieta JK, Smith YR, Bueller JA, Xu Y, Kilbourn MR, Jewett DM, Meyer CR, Koeppe RA, Stohler CS. Regional mu opioid receptor regulation of sensory and affective dimensions of pain. Science 2001; 293:311–5. Reprinted with permission from AAAS.

Zubieta et al.^72^ also demonstrated a substantial interindividual variability in both receptor availability and in the activation of the mu-opioid system in healthy controls experiencing same level of pain intensity. In these, and subsequent studies, the pain intensity was controlled (moderate pain was kept constant at around 40 in the visual analogue scale throughout the whole period of the experiment) (Fig. 2). These results added important data, which, at least in part, explains the very large variability in pain experience across individuals. This variability embodies a fundamental consideration when deciding the most adequate therapeutic approach to treat each patient with chronic pain. Interindividual variability in mu-opioid activation was also implicated in the vicarious pain study and very likely accounts for the differences in the reactions of seeing others in pain, which were clearly detected among individuals.^29^ Furthermore, the variability in individual responses to pain, as well as in the functioning of the endogenous antinociceptive mechanisms, has also been attributed to genetic factors. This information is paramount from the perspective of clinical pain management, particularly when evaluating individual susceptibilities to chronic pain development, the prediction of treatment outcomes, and the choice of the most appropriate therapies to treat each subject (individualized treatments). Indeed, genetic variants may not only interfere in the protein coded by a gene but also in the physiological activity of several neural systems, including the opioidergic system. Thus, the role of genetic polymorphisms on the antinociceptive activity of the opioid system has also been examined through PET.

**Figure 3.**
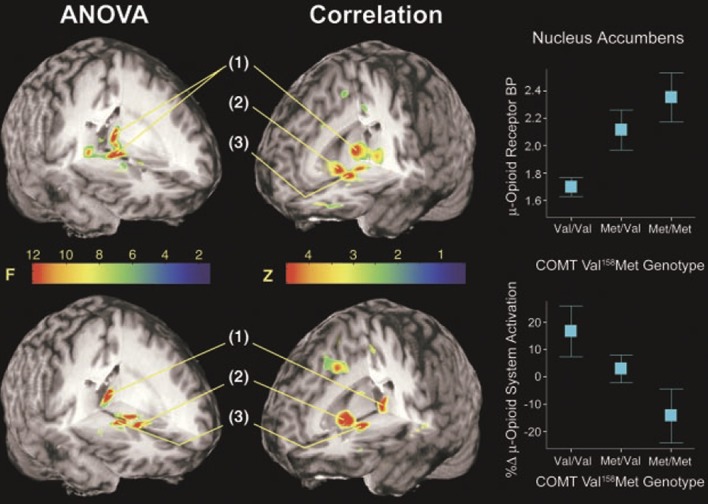
Effects of COMT *val^158^met* genotypes and related COMT activity on the mu-opioid system activation induced by sustained pain. Upper left: effects of COMT *val^158^met* genotypes on baseline mu-opioid receptor availability. Significant effects on baseline were observed in the anterior and posterior (pulvinar ipsilateral to pain) thalamus (1). Near the multiple comparisons threshold, possible effects were observed bilaterally in the NAc and ventral pallidum and in the contralateral thalamic pulvinar. Lower left: effects of COMT *val^158^met* genotypes on the activation of the mu-opioid system during sustained pain stress. Significant effects were observed in the anterior and posterior (pulvinar) thalamus (1) and striatopallidal regions (NAc (2), ventral pallidum (3), and subthalamic nucleus, bilaterally). Upper right: correlations between COMT activity related to the COMT *val^158^met* polymorphisms and baseline mu-opioid receptor availability in the NAc COMT activity was coded as follows: −1, *met/met*; 0, *val/met*; and 1, *val/val*. Lower right: correlations between COMT activity and mu-opioid system activation in response to the pain challenge in the NAc.^74^ From Zubieta JK, Heitzeg MM, Smith YR, Bueller JA, Xu K, Xu Y, Koeppe RA, Stohler CS, Goldman D. COMT val158met genotype affects mu-opioid neurotransmitter responses to a pain stressor. Science 2003;299:1240–3. Reprinted with permission from AAAS. ANOVA, analysis of variance; BP, binding potential; COMT, catechol-O-methyltransferase; NAc, nucleus accumbens.

Early evidence regarding the genetic component of pain processing comes from a PET study that used a selective opioid mu-opioid receptor radiotracer to investigate the effects of the abundant polymorphism of the catechol-O-methyltransferase (COMT) gene, which codes the replacement of valine (val) by methionine (met) at the codon 158 (*val^158^met*), on the mu-opioid activation induced by a sustained pain challenge^74^ in healthy subjects. Since COMT is one of the enzymes that metabolizes catecholamines, it is involved in the regulation of noradrenergic and dopaminergic systems and may indirectly affect opioid neurotransmission. This study reported that the mu-opioid activation induced by the sustained pain challenge was significant lower in *met/met* healthy subjects, which have a lower COMT activity, when compared with *val/met* subjects, which present an intermediate function of the same enzyme. Those differences were found in the striatopallidal pathway nuclei and amygdala^74^ (Fig. 3). These COMT polymorphisms (*val^158^met*) were also accompanied by higher sensory and affective ratings of pain in healthy subjects. A more recent study has confirmed that in chronic temporomandibular disorder pain, COMT polymorphisms (*met/met-met/val*) have induced higher pain sensitivity, but with changes in mu-opioid activity in the limbic system. Hence, further studies should be performed to better understand the effect of COMT polymorphism and mu-opioid activity in multiple pain conditions and disorders.^44^

Other polymorphisms affecting the activity of the mu-opioid system have also been studied. For instance, the *A118G* polymorphism of the mu-opioid receptor is a single nucleotide polymorphism characterized by the substitution of adenine (A) to guanine (G) at codon 118 (*A118G*) in the human *OPRM1*. This is related to a lower expression of the *OPRM1* gene.^69^ Hence, it interferes with pain sensitivity as well as the outcomes of pain treatments, with important effects on the modulatory activity of the opioid system. This polymorphism has been recently investigated in clinical pain conditions.^66^ For example, patients with primary dysmenorrhea were evaluated through functional MRI (fMRI). The results showed that the individuals who carried the G allele had functional hypoconnectivity between the ACC and periaqueductal gray matter (PAG) when compared with AA homozygotes, which also correlated with the spontaneous menstrual pain. Patients with primary dysmenorrhea carrying the G allele exhibited no correlation between PAG-seeded FC and their menstrual pain. These findings indicated a decreased opioid modulation of pain in G allele carriers. The results of that study also brought important insights into the mechanisms of pain in dysmenorrhea, and such data help to explain the great individual variability in the pain experience reported by those patients. A later study reported that other genetic variations in the mu-opioidergic system, more specifically, the presence of the T-allele of the rs563649 polymorphism on the mu-opioid receptor, may influence the prediction of the development of pain complaints at the ages of 16 to 17 years, from behavioral and functional indicators (eg, reward feedback–related response in the ventral striatum and PAG) obtained at earlier ages (14–15 years).^46^

A negative correlation has also been demonstrated between the sensory features of pain, evaluated through the sensory score of the McGill Pain Questionnaire (MPQ) and the degree of mu-opioid activation in the thalamus, NAc, and amygdala using the model of experimentally induced sustained pain previously described.^73^ The presence of the same correlation in the PAG, although not reaching the statistical level of significance, assigns an antinociceptive function to the amygdala, possibly through its connections to the PAG, a region with a dense concentration of mu-opioid receptors and an essential element of the descending pain modulatory system.^40^ The correlation between the NAc mu-opioid activation, a key component of the reward circuitry^21^ with the sensory pain ratings found in the same study, indicates the contribution of this structure to the antinociceptive effects produced by mu-opioid receptor activation. In addition, significant negative correlations were found between MPQ affective scores and mu-opioid receptor activation in the NAc as well as in the ACC and thalamus. The changes in thalamic mu-opioid activity are also relevant. A recent fMRI-PET study demonstrated a colocalization of changes in opioid receptor availability measured with [11C] diprenorphine and the hemodynamic changes in the brain obtained through changes in the BOLD signal within the thalamus, mediated by noxious pressure stimulus in healthy subjects.^67^ These findings were interpreted as a possible inhibition of thalamic neurons by a release of endogenous opioids induced by the painful stimulus.

Taken together, these results suggest that the antinociceptive effects of mu-opioid receptor activation by endogenous opioids are not only important to reduce the sensory aspects of pain but also to relieve its affective features. Such results also opened possibilities to investigate the relationship between mu-opioid receptors and their relationships with other systems important to pain and inflammation, which was explored in a following study.

Using the model of sustained pain previously described, a PET study revealed a significant negative correlation between the baseline plasma levels of the proinflammatory cytokine interleukin 1β (IL-1β) and the availability of mu-opioid receptors in the amygdala. In that study, subjects with higher levels of IL-1β showed lower mu-opioid receptor availability in the amygdala along with higher pain sensitivity.^52^ On the other hand, during a pain challenge (sustained pain applied to the masseter muscle), the activation of mu-opioid receptors was positively correlated with the plasma levels of Il-1ra (antinociceptive cytokine), but not with the plasma levels of IL-1β in the dorsomedial NAc. Nevertheless, the analgesic effects produced by the mu-opioid system activation during the pain challenge were counterbalanced by changes in IL-1β. For instance, subjects that displayed higher pain-induced release of IL-1β experienced less opioid-induced analgesia. The effects of IL-1β in the functioning of the opioidergic system have also been demonstrated in in vitro and in animal model studies. For instance, in a past study, IL-1β promoted an increase in the expression of proenkephalin and opioid receptors mRNA in primary astrocyte-enriched cultures.^54^ Furthermore, the release of IL-1β in the peripheral ganglia has been proposed as a possible mechanism to the attenuation of the analgesic effects related to morphine, a process that possibly involves the activation of satellite glial cells in the peripheral ganglia.^9^ In fact, bidirectional interactions between peripheral inflammation and central mechanisms as well as their contributions to the development of pathological conditions had been largely reported.^4^ Corroborating this concept, changes in the functioning of the opioid system have been found in patients with inflammatory pain.^28^ In addition to mechanistic studies, the contribution of the opioid system to pain has been extensively investigated through PET in cohorts of patients with episodic/chronic pain, and the role of mu-opioid activation in the pathophysiological mechanisms of different painful syndromes has just started to be unveiled.

## 3. Changes in the opioid system in painful syndromes and chronic pain

The results of MRI studies indicate that chronic pain is associated with structural changes in the central nervous system. Such changes include altered cortical thickness, eg, cortical thickening or cortical thinning, previously demonstrated in patients with migraine^15,24^ or changes in the cortical volume, eg, decrease cortical volume found in a cohort of patients with back pain.^5^ More importantly, those changes have been proven to affect multiple cortical structures involved not only in pain perception but also in the modulation of nociceptive stimuli. In addition, fMRI studies suggest the occurrence of colocalized functional and structural changes in the brain of patients with chronic pain.^14^ Nevertheless, beyond the presence of structural and metabolic changes in the brain induced by a persistent pain experience, PET studies have brought insights into the molecular neuromechanisms underlying the maladaptive neuroplasticity related to chronic pain. The use of PET in pain research has also helped researchers understand the individual viability related to pain, as well as long-lasting resistance to treatment, including to opioids found in refractory patients. Recent studies with (PET) using nonselective (eg, [11C] diprenorphine) radiotracers or [11C] carfentanil, a selective mu-opioid receptor radiotracer, have shown reduced mu-opioid receptor availability in some chronic pain disorders, including rheumatoid arthritis,^28^ neuropathic pain,^37^ and fibromyalgia.^25^

The first evidence of changes in the opioid system in patients with chronic pain dates back to the 1990s. A preliminary PET study performed in 4 patients with rheumatoid arthritis and using [11C] diprenorphine showed increased binding related to pain relief in several brain regions. However, more significant changes were reported in the temporal, frontal, and cingulate cortices.^28^ Similar results were obtained in a sample of 5 patients with central poststroke pain. Those patients presented lower opioid receptor availability in several pain-related brain structures, such as the thalamus, anterior and posterior cingulate cortex, midbrain, insula, PFC, S2, and parietal cortex, when compared with a control group of healthy subjects.^68^ Although still preliminary, those studies evidenced the presence of an altered opioid neurotransmission in chronic pain and highly suggested that a decreased availability of opioid receptors could be a common finding in subjects with chronic pain. In another PET study conducted in patients with arthritis (osteoarthritis, n = 15 and rheumatoid arthritis, n = 2) using [11C] diprenorphine, higher chronic pain levels were associated with increased availability of opioid receptors in the insula, PAG, basal ganglia, NAc, and subcallosal area in the group of patients, which could suggest upregulation of opioid receptor sites in patients with chronic pain who present higher levels of pain.^10^ The availability of opioid receptors in the caudate nucleus was also positively correlated to thermal pain thresholds in both patients and healthy subjects, supporting the role of the upregulation of opioid receptors within the striatum as an adaptive response to inhibit pain. Finally, the same study showed reduced levels of opioid receptor availability in the caudate nucleus of patients with chronic pain compared with controls, supporting the findings of the other studies. Nevertheless, a common limitation of those studies was the use of a nonselective radiotracer, which did not permit the identification of the specific opioid receptor affected by chronic pain.

In another work, Harris et al.^25^ used the selective mu-opioid receptor radioligand [11C] carfentanil to study the functioning of the mu-opioid system in fibromyalgia syndrome, a disease with unclear etiology and pathophysiology. The results indicated a reduced availability of mu-opioid receptors in the amygdala, NAc, and dorsal ACC of 17 patients with fibromyalgia when compared age- and sex-matched healthy controls. When analyzing the group of patients with fibromyalgia separately, the availability of mu-opioid receptors in the striatum and cingulate cortex was negatively correlated with the relative amount of affective pain, defined by the affective/sensory scores of the MPQ. Furthermore, the availability of mu-opioid receptors in the NAc was negatively correlated to the affective pain ratings. When expanding the findings obtained with fibromyalgia to other chronic pain diseases, reduced availability of mu-opioid receptors in the NAc was also found in a cohort of patients with trigeminal neuropathic pain.^19^ Those changes were also correlated with the clinical pain symptoms. Nonetheless, in the cohort of patients with trigeminal neuropathic pain, the availability of mu-opioid receptors in the NAc was negatively associated with the sensory and total pain ratings of the MPQ. The NAc is the main structure in the ventral striatum. Although very small, this brain region plays a crucial role in the reward circuitry and modulation of the nociceptive information. It receives afferent nociceptive inputs through connections with the amygdala, thalamus, cingulate cortex, and parabrachial nucleus. Moreover, the axis formed by the PAG, rostralventromedial medulla, and NAc exerts a crucial role in the descending pain modulatory system.^40^ The analgesic effects of several analgesic drugs, including opioids, highly depend on the proper functioning of those structures.^2^ Remarkably, changes in the BOLD signal of the NAc has been related to the onset (aversive) and offset (reward) of painful heat stimuli.^7^ However, even more important seems to be the changes in activity of the NAc in response to the offset phase of painful heat stimuli, which permitted the differentiation between patients with chronic low back pain and healthy subjects in a previous study.^6^

A PET/fMRI study performed in patients with fibromyalgia revealed a positive relationship between pain-evoked neural activity and mu-opioid receptor availability in the rostral ACC (rACC) and in the dorsolateral PFC (DLPFC). On the other hand, fMRI BOLD signals in several brain regions such as the medial frontal gyrus, posterior cingulate cortex, and ACC were negatively correlated with the affective/sensory pain ratio. Furthermore, higher mu-opioid receptor availability was negatively correlated with the affective/sensory pain ratio in the medial frontal gyrus, posterior cingulate cortex, ACC, and precentral gyrus.^56^ Based on such findings, the authors hypothesized that in patients with fibromyalgia, tonic higher levels of endogenous opioids would promote a lower affinity or a downregulation of mu-opioid receptors on GABAergic interneurons of brain regions involved in nociception. Hence, an inhibition of GABAergic interneurons related to a phasic release of endogenous opioid triggered by noxious stimulation, an important mechanism for the descending pain modulation, could be altered in fibromyalgia. More specifically, although in healthy individuals, opioid release results in the inhibition of GABAergic interneurons that tonically inhibit the neurons located in several brain regions such as the PAG, PFC, and ACC, thus causing disinhibition and excitation of antinocicpetive neurons, in patients with fibromyalgia, such phasic endogenous opioids release in response to nociceptive stimuli fails in producing adequate inhibition of GABAergic interneurons and a faulty antinociceptive mechanism occurs.^56^

The activity of mu-opioid receptors has also been studied in patients with episodic migraine during both its ictal (painful) and interictal (pain-free) phases. Increased activity of mu-opioid receptors in the PFC was reported during the ictal spontaneous migraine phase compared with interictal periods.^16^ Indeed, PFC activation had been previously shown in both triggered and spontaneous migraine headache attacks.^1,18^ Moreover, it seems that an increased mu-opioid activity in the PFC enhances the connectivity with the PAG, which is important to produce analgesia.^65^ In addition, the mu-opioid activity during the ictal and interictal phases of patients with migraine was positively correlated, corroborating the interindividual variability in the mu-opioid system largely reported and already discussed in this article.^73^ In a following PET study also performed in patients with episodic migraine, the activity of the mu-opioid neurotransmission in the red nucleus and PAG was positively correlated to the development thermal allodynia in an experimental paradigm using a heat stimulus applied to the ophthalmic (V1) region of the trigeminal nerve.^45^ The results of that study substantiate the concept of a dysfunctional response of midbrain structures to the nociceptive inputs received from activated afferent trigeminal projections as a mechanism of migraine headaches.^30,61^ Conversely, a recent PET study did not find differences in the availability of opioid receptors when comparing patients with episodic migraine and healthy subjects.^34^ However, the lower sensibility of diprenorphine,^53^ used to evaluate the functioning of the opioidergic system in that study along with the comparison at baseline conditions (eg, patients with migraine were scanned within their interictal phase), might explain their distinct results when compared with the previous studies.^16,45^

As previously mentioned, the findings of these studies could indicate a higher release of endogenous opioids related to pain or downregulation of opioid receptors, particularly mu-opioid receptors, or even both. Those hypotheses have only been recently tested by a PET study performed in an experimental animal model of neuropathic pain.^62^ The authors scanned Sprague-Dawley rats with [18F] FDPN, a compound analog of diphrenorphine. Animals were scanned 3 months after the spared nerve injury or sham surgery was performed. Animals that underwent the spared nerve injury exhibited lower availability of opioid receptors in the motor cortex and basal ganglia when compared with sham. Although a baseline scan was not performed, the observed changes were attributed to the presence of neuropathic pain, since all animals were exposed to the same experimental conditions. More importantly, the same study found a decreased expression of mu-opioid receptors in the basal ganglia and insula of the animals that developed a peripheral neuropathy, which was not followed by changes in enkephalin or in the neuronal marker NeuN, as demonstrated by immunohistochemistry. Considering those findings, the decreased availability of opioid receptors observed in the brain of patients with chronic pain might be interpreted as variations in the expression of opioid receptors triggered by pain rather than loss of opioidergic neurons (no changes in NeuN-immunoreactivity cell count between nerve-injured and control rats) or higher release of endogenous opioids (no difference in enkephalin immunoreactivity between nerve-injured and control rats).^35^ Nevertheless, the lack of a longitudinal evaluation (eg, preneuropathic and postneuropathic pain) is an important limitation that should be addressed in future studies. Moreover, to which extent those findings can be translated to other (non-neuropathic) chronic pain disorders, as well as to humans, are important aspects that must considered when analyzing such results. The use of selective radiotracers by future studies will also be important confirm the specific opioid receptor affected in this whole process. In addition, another important feature that has been scarcely explored and should be investigated in depth by future studies is the difference in opioid activation between somatic and visceral pain. For instance, at least 1 study reported that in contrast to somatic pain, sustained visceral pain is not related to an increased release of mu-opioid receptors, despite similar intensities, thus highly suggesting that endogenous opioids could play distinct roles in each type of pain (eg, somatic or visceral pain).^36^

## 4. Placebo-nocebo and the opioid system: an overview of the evidence obtained from positron emission tomography studies

The opioid system has been widely associated with the development of placebo effects since the discovery that that the placebo effect can be prevented by the administration of naloxone, an opioid receptor antagonist.^33^ Further studies demonstrated the central pathways involved in the placebo effect. This includes cortical areas, such as the ACC and DLPFC, the descending modulatory system components (eg, PAG and rostroventromedial medulla), as well as the dorsal horn of the spinal cord.^22,51,64^ In addition, the neurobiology of placebo and nocebo effects has been investigated through PET studies. Overall, these works have confirmed, in humans, the fundamental role of the dopaminergic and opioidergic system on placebo/nocebo as well as the specific areas engaged in the processing of both phenomena, mainly the reward circuitry with its functioning centered in the activity of the NAc. This progress in the understanding of the precise neuromechanisms underlying placebo and nocebo effects can potentially impact the evaluation of clinical trial results, development of therapeutic strategies, and even from a broader perspective, the comprehension of the interindividual variability related to pain experience. The activation of the mu-opioid system in the placebo response was demonstrated in an early PET study. Significant activation of the mu-opioid system was found in the insula, rACC, DLPFC, and NAc.^70^ More importantly, the mu-opioid system activity was correlated with the pain experience. For instance, the DLPFC mu-opioid activation found in that study was related to the degree of the expected analgesia induced by placebo. Interestingly, the same study applied a system for pain delivery that allowed the measurement of variations in the requirements of the algesic substance used before and after the placebo administration. Therefore, it provided an objective evaluation of changes in pain sensitivity induced by placebo, or in other words, the activation of antinociceptive mechanisms. Mu-opioid activation in the rACC was highly correlated with the requirements of the algesic infusion to keep the pain during the study.^70^ In fact, the role of rACC in the placebo mechanisms has been shown by other studies.^3,22^ It has also been demonstrated that the coupling between the ACC and PAG induced by placebo can be blocked by naloxone.^22^

The investigation of the role of expectations and learning processes to the activation of the mu-opioid system during placebo has revealed that the prediction error signal (eg, the difference between the expected and perceived analgesia) is related to the analgesic responses produced by placebo and to the mu-opioid activation in the ACC, insula, amygdala, thalamus, and OFC.^50^ In the same study, individuals with low expectations and higher effectiveness (representing a positive prediction error signal) exhibited the strongest placebo responses, whereas subjects with higher expectations and lower effectiveness (eg, negative prediction error signal) displayed nocebo responses.

A further study investigated compared the roles of the opioidergic and dopaminergic systems with the development of placebo and nocebo effects in healthy volunteers.^59^ Remarkably, nocebo and placebo produced opposite effects in those systems. Activations of both dopaminergic and opioidergic systems were found with placebo while deactivations occurred with nocebo. Placebo-induced activation in the dopaminergic system was found in the basal ganglia, including the NAc. Indeed, the dopaminergic release in the NAc was responsible for 25% of the variance in the analgesic effects produced by placebo. In the opioidergic systems, those effects were also detected in the NAc but extended to other brain regions such as the PAG, amygdala, OFC, insula (anterior and posterior), and ACC (rostral and subgenual). On the other hand, 25% of the sample experienced hyperalgesia with the administration of the placebo, which represented the nocebo effect. Nocebo correlated to deactivation of the dopaminergic and opioidergic systems with plentiful overlap of the brain regions affected by both effects (placebo and nocebo). When comparing placebo-induced changes in the activity of mu-opioid and dopamine neurotransmissions between high placebo and nocebo responders, again, significant deactivations were found in nocebo responders opposing to the activations found in the group of placebo responders. Regarding the mu-opioid neurotransmission, the changes were found in the subgenual ACC, anterior insula, OFC, and thalamus, while in the dopaminergic system, similar findings were detected in the NAc and putamen. Another important finding of that study was that the degree of placebo-induced dopaminergic and opioidergic activations in the NAc predicted high and low placebo responsiveness. Furthermore, the activation of dopamine neurotransmission in the NAc correlated with the degree of mu-opioid activation in regions that responded to placebo. Such findings corroborate the importance of dopamine receptors to the activity of mu-opioid neurotransmission^12^ as well as the participation of mesolimbic dopaminergic projections and its interactions with the opioidergic system to the placebo analgesia.^58^ In fact, it has been described that placebo characterizes a type of reward expectation; therefore, both share the same pathways.^17^

The activation of the mu-opioid system induced by placebo has also been investigated from the perspective of specific clinical conditions and alternative pain treatment modalities. One of those studies investigated the effects of a placebo treatment in patients with episodic migraine during their interictal phase using PET with [11C] diprenorphine. Although the placebo treatment resulted in decreased pain scores, the magnitude of the placebo effect was not significantly different between placebo and controls. Moreover, patients with migraine and healthy controls did not differ regarding activity of opioid receptors induced by placebo.^34^ As expected, few subjects from each group reported higher pain levels with the placebo treatment. Those individuals were classified as nocebo responders instead of placebo responders. Another study investigated the neuromechanisms related to a method of noninvasive modality of neuromodulation, namely transcranial direct cranial stimulation (tDCS) on healthy subjects through PET.^20^ The results demonstrated that sham and real stimulation (2 mA for 20 minutes) induced mu-opioid activation in shared (precuneus and PAG) as well as in specific brain regions (the thalamus for sham and the PFC for real tDCS). These data support the presence of a placebo effect contributing to the clinical effects of tDCS. Moreover, those findings suggest that both shared and specific mechanisms underlie the effects of such method. Similar findings have also been demonstrated with other neuromodulatory techniques, such as transcranial magnetic stimulation (TMS), confirming the activation of the mu-opioid system driven by noninvasive methods of neuromodulation.^31^ More specifically, in a study published by Lamusuo et al.,^31^ the availability of mu-opioid receptors was lower after real TMS applied to S1/M1 when compared with sham stimulation in the right ventral striatum, medial orbitofrontal, prefrontal and anterior cingulate cortices, left insula, superior temporal gyrus, dorsolateral PFC, and precentral gyrus. These results might represent a higher release of endogenous opioids driven by TMS. Another study assessed the availability of opioid receptors, measured through the nonselective radiotracer [11C] diprenorphine, preoperatively and postoperatively, in patients with neuropathic pain treated with motor cortex stimulation (MCS). The results indicated that the levels of opioid receptors assessed through preoperative PET scans may be valuable predictors of MCS outcomes when used to treat neuropathic pain. For instance, in that particular study, patients exhibiting values of opioid receptor density below the lower limits in age-matched controls in the contralateral insula, PAG, and thalamus were presented the lowest probabilities to benefit from MCS treatment.^38^ An additional PET study also reported a focal release of endogenous opioids driven by deep brain stimulation (DBS) of the PAG. However, those effects were neither affected by the systemic administration of the opioid antagonist naloxone nor correlated to the analgesic effects produced by DBS. Therefore, such findings were not able to determine whether the analgesic effects produced by DBS are indeed driven by a DBS-related opioid release.^60^ Another study that explored the effects of traditional and sham (placebo) Chinese acupuncture in patients with fibromyalgia^26^ also suggested differential mechanisms, both involving the mu-opioid neurotransmission, underlying each one.

Interestingly, some studies have indicated that placebo effects and the related mu-opioid activation may be predicted by some personality traits^48^ and influenced by genetic factors.^49^ Such information could be extremely useful when designing clinical trials, especially in cases of therapies previously expected to produce large placebo effects. The use of such information would permit the subdivision of clinical pain study samples based on the potential development placebo/nocebo effects and the presence of genetic polymorphisms or personality traits that exert direct influence over the placebo response. Another fact still scarcely explored in the literature is how the degree of placebo responses can predict the effectiveness of nonpharmacological therapies such as cognitive-behavioral therapy or neuromodulatory therapies.^51,55^

## 5. Conclusions and perspectives

The possibility of studying the human opioid system in vivo has contributed important information to the understanding of the antinociceptive mechanism related to acute and chronic pain, as well as the neurochemical basis of both placebo and nocebo effects. The altered functioning of the opioid system in the brain of patients with chronic pain represents one of the most important findings obtained with clear therapeutic implications. The interindividual variability in the mu-opioid system activation is another prominent result provided by PET studies. Future studies are expected to expand the overall knowledge regarding the contribution of genetic polymorphisms to the differential responses of the mu-opioid system to pain and to the individual resiliency or susceptibility to chronic pain development. The use of that information, together with the investigation of genetic, potentially inflammatory markers, or even associated personality traits, will be extremely useful in the development of clinical trials with better stratification of the individuals, analyzed based on their expected activation of the mu-opioid system as well as on their predisposition to experience placebo or nocebo effects.

## Disclosures

The authors have no conflict of interest to declare.

A.F. DaSilva is funded by the National Institute of Health–National Institute of Neurological Disorders and Stroke—R01 NS094413, and National Institute of Dental and Craniofacial Research—U01 DE025633. M.F. DosSantos is funded by Fundação Carlos Chagas Filho de Amparo à Pesquisa do Estado do Rio de Janeiro (FAPERJ), Jovem Cientista do Nosso Estado (JCNE), and Conselho Nacional de Desenvolvimento Científico e Tecnológico (CNPQ).
